# Estimating waves via measured ship responses

**DOI:** 10.1038/s41598-023-44552-2

**Published:** 2023-10-13

**Authors:** Ulrik D. Nielsen, Harry B. Bingham, Astrid H. Brodtkorb, Toshio Iseki, Jørgen J. Jensen, Malte Mittendorf, Raphaël E. G. Mounet, Yanlin Shao, Gaute Storhaug, Asgeir J. Sørensen, Tomoki Takami

**Affiliations:** 1 Department of Civil and Mechanical Engineering, Technical University of Denmark, 2800 Kgs. Lyngby, Denmark; 2 Department of Marine Technology, Norwegian University of Science and Technology, 7491 Trondheim, Norway; 3 Tokyo University of Marine Science and Technology, Etchujima, Tokyo, 135-8533 Japan; 4 DNV, 1363 Høvik, Norway; 5 National Maritime Research Institute, Shinkawa, Tokyo, 181-0004 Japan

**Keywords:** Mechanical engineering, Physical oceanography

## Abstract

Optimisation of energy efficiency and operational performance as well as assessment of safety levels and emissions of marine operations require detailed information about the acting wave system. It is possible—with an analogy to classical wave buoys—to estimate the directional wave spectrum by processing sensor measurements of wave-induced responses (e.g., motions and structural responses) from a ship. Compared to other sources of wave data (e.g., buoys, satellites, third-generation wave models), estimation concepts using the ship itself as a buoy provide the wave spectrum at the exact spatio-temporal point, potentially increasing accuracy and with minimal associated cost. This paper gives an overview of the technology, discusses associated uncertainties, and highlights new developments made for estimating waves via measured ship responses.

## Introduction

**Figure 1:**
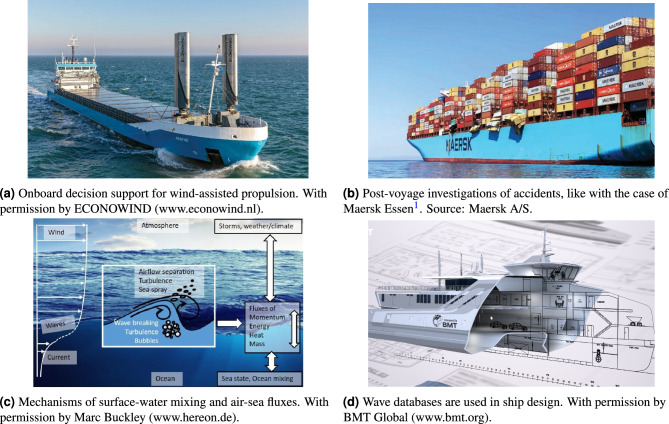
Measuring ocean waves. Information about waves is of crucial importance in many applications considered by engineers and ocean scientists.

*Measuring* ocean waves is of fundamental importance to shipmasters, naval architects, maritime engineers, and ocean scientists alike, since waves are the basic driver of most of the processes of their concern. Here, we note that measuring ocean waves is equivalent to measuring the directional wave spectrum, representing the energy density of the wave components forming the wave system^1^. Applications dependent on measurements and a characterisation of waves are, e.g.: (a) analysis of wave-structure interactions before/during/after operations, (b) collection of historical data for design and rule specification for offshore structures and ships, (c) preliminary evaluation of offshore sites for renewable energy potential and construction, and (d) assessment of mechanisms of surface-water mixing and air-sea fluxes for understanding weather and climate changes. Examples of use-cases are illustrated in Fig. 1, where the following additional remarks can be added: For the optimal operation of ships sailing in a seaway, real-time, onboard decision support systems can guide the crew to select speed and heading to ensure high levels of safety and performance. For instance, for vessels operating with wind-assisted propulsion, as illustrated in Fig. 1a, it is difficult to decide on a heading where the thrust from wind is maximised while, at the same time, keeping the added resistance from waves minimum. Such a heading selection can be guided by a decision support system if the directional wave spectrum is available. Similarly, decision support systems can warn against the risk of being in dangerous situations, where large motions or parametric roll can lead to loss or damage of cargo^2^, as illustrated in Fig. 1b. It may also be that an incident, having resulted in container damages and losses, needs further *post*-voyage investigations, e.g., for reasons of insurance, in which case the in-situ directional wave spectrum must be available. Thus, with the green transition and digitalisation period we are in, soon all vessels need some kind of wave estimation technology for reasons of safety, emission reductions, and inspection. Lastly, it is noted that the development of weather and storms is largely affected by the exchange of energy, momentum, and mass between the atmosphere and the sea, cf. Fig. 1c^3^. This exchange also influences the carbon budget of the ocean and atmosphere, and thus with a crucial importance to mechanisms related to climate change^4,5^. At a fundamental level, the exchange between air and sea takes place in the boundary layers over the sea surface, and variations of the surface, characterised by waves, should therefore be measured to enable understanding and modelling of weather and climate.

**Figure 2:**
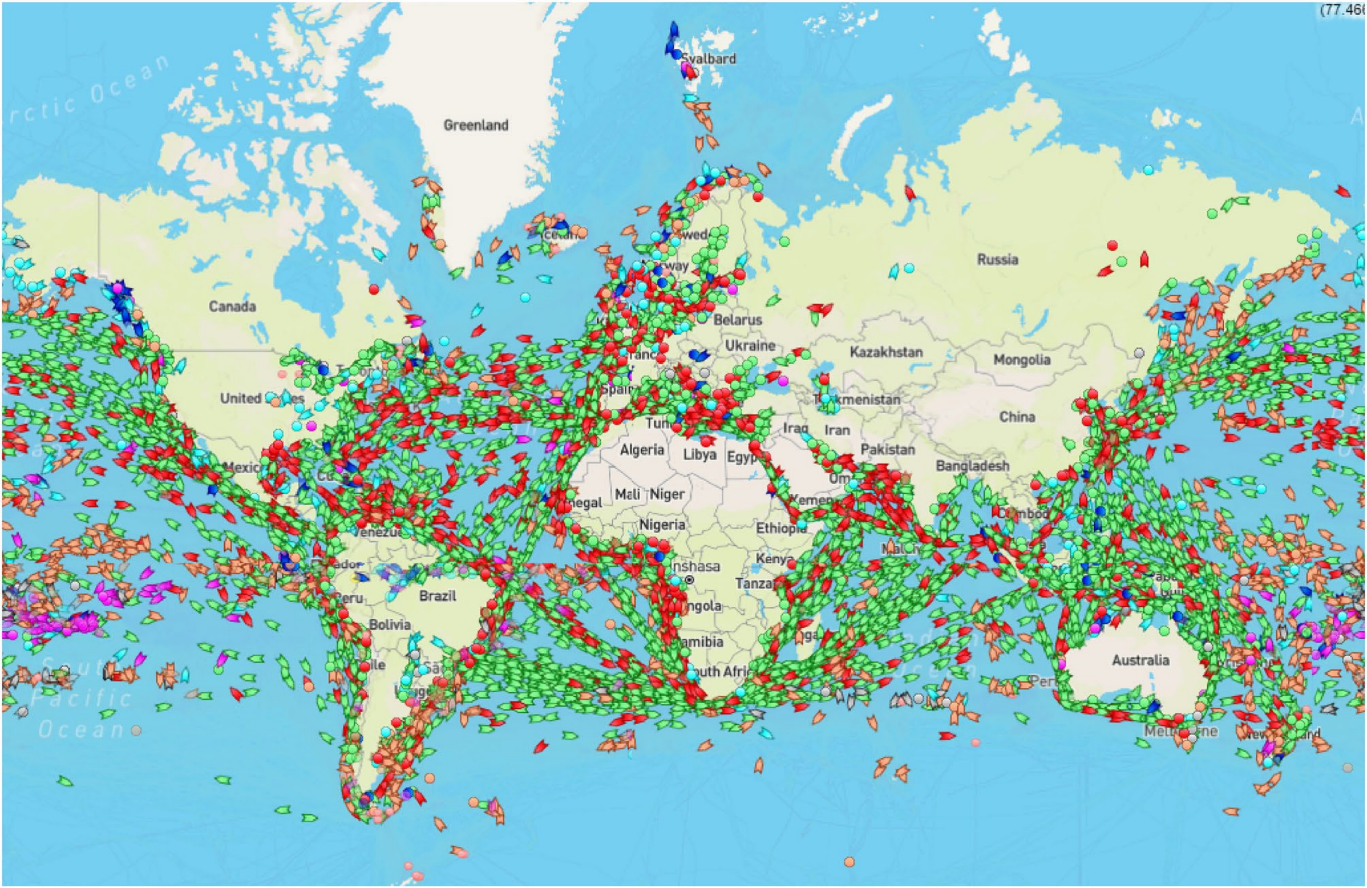
Snapshot of vessel positions, as of 27-06-2023 UTC 10:30, from AIS data with colour codes for identification of ship type, noticing that more than 300,000 ships appear. With permission by MarineTraffic (www.marinetraffic.com).

In practice, waves are typically *estimated* by a buoy, where the buoy’s translational and angular motions, resulting from waves, are the basis for the estimate. By analogy it is possible to use recorded wave-induced motions of a ship to estimate waves. This latter principle, referred to as the wave buoy analogy or ship-as-a-wave-buoy, has been investigated and developed over the past 2-3 decades with a handful of pioneering studies^6–10^. Generally, the concept is well-understood and produces fair to good results compared to real (“classic”) wave buoys, despite the complexities inherent due to a ship’s large size (length, breadth, draught), relative to the waves we are trying to measure, making a ship behave as a low-pass filter, and since a ship typically advances in the propagating waves. On the other hand, compared to buoys - being deployed in fixed locations, and extremely scarcely located considering the coverage of the oceans - there is an obvious but unexploited potential in using ships as *sailing* wave buoys. Figure 2 highlights the potential by imagining any ship to be a sailing wave buoy. Hence, accounting for the sheer number (+300,000) of ships in operation, the spatial and temporal coverage considering wave estimation and other environmental data is significant, assuming mechanisms and routines for data sharing.

### Scope and novelty

This paper gives an overview and presents new developments made at the Department of Civil and Mechanical Engineering, Technical University of Denmark, with selected partners for *estimating waves via measured ship responses*. Onward, we refer to wave-estimation technologies of this type under the umbrella term the Wave Buoy Analogy (WBA). In the paper, we present new results of a novel framework consisting of a hybrid method^11^ combining physics-based and machine learning-based wave estimates. Specifically, we introduce an experimental dataset obtained under “controlled conditions” during sea trials, and we show that reasonable wave estimates can be expected using a physics-based method under such favourable conditions. In contrast, for a ship in service, the level of uncertainties associated with operational parameters such as speed and draft can be significant. This motivates the development of a hybrid method, being less sensitive to the aforementioned uncertainties and thus more robust. In concrete terms, the hybrid framework is realised by conditioning the physics-based method on results, i.e. wave estimates, from another source, practically achieved by running a machine learning model concurrently. The hybrid method, as a concept and with its theoretical derivations, has been presented in an earlier paper^11^ where the machine learning model was trained using data from a wave radar. In the present work, we introduce a new set of results, derived by training the machine learning model instead by the ERA5 database^12^, to facilitate the conditioning. The advantage in training the machine learning model with ERA5 is that this approach does not require any (extra) hardware on the ship, which for instance is the case if a wave radar should be installed.

## The *Wave buoy analogy* in a nutshell

**Figure 3:**
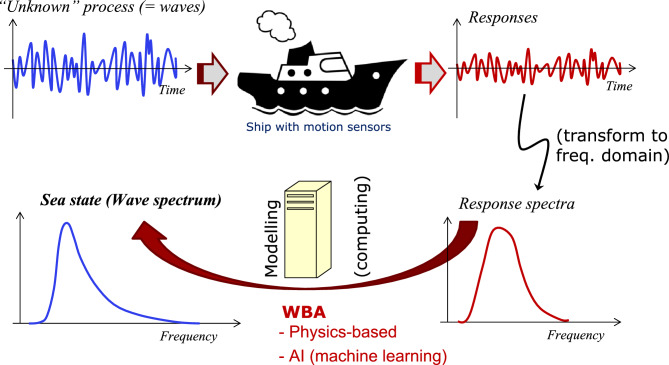
Sketch of the wave buoy analogy (WBA), alluding to frequency-domain frameworks.

Fundamentally, the WBA relies on measuring wave-induced ship responses; say, heave (*z*), roll ($\phi$), and pitch ($\theta$). The principle is sketched in Fig. 3. Introducing the linear time-invariant (LTI) assumption facilitates the use of wave-to-motion transfer functions which leads to what will be referred to as physics-based methods with formulations in both the frequency domain and the time domain. In contrast thereto, purely data-driven approaches exist, relying on machine learning. The main advantage of physics-based methods is that they can provide estimates of the complete directional wave spectrum, where different mathematical strategies^13–26^ are applied for solving the highly underdetermined equation system describing the physical relationship between the energy densities of waves and the corresponding responses. In machine learning methods^27–36^, the general relationship between wave *parameters* and induced responses of a particular ship is learned by comprehensive training with large datasets of measured responses against available sea state information, e.g. from hindcast wave databases. The main advantage of machine learning methods is that transfer functions are not needed, and all uncertainties associated with transfer functions are thus removed. On the other hand, the need for high-quality sensor measurements is emphasised and so is the importance of having accurate (“external”) data used as a proxy for the true wave parameters at the exact spatio-temporal position of the ship. Besides, machine learning methods offer, presently, only wave parameters (not the detailed wave spectrum), although research has been initiated by the international community to relax this restriction, as discussed later.

### Hybrid framework

The state of the art literature, as introduced above, demonstrates that machine learning methods, in fact, tend to give better estimates of the wave parameters compared to physics-based methods, when full-scale, operational data from in-service ships is considered. It is thus interesting to compose a hybrid framework, combining results from a machine learning method with the capability of a physics-based method, providing the detailed directional wave spectrum. Such a framework has recently been developed by establishing an algorithm^11^ that outputs a directional wave spectrum which is conditioned on results from a machine learning method. More specifically, the hybrid framework takes estimated wave parameters from a convolutional neural network^32^ and uses them as constraints on the directional wave spectrum subsequently computed by a physics-based method^8,13^. It is noteworthy that the specific physics-based method relies on Bayes’ theorem^37^, and, in the following, we will refer to it as the *Bayesian method*.

## Results

The potential of a ship as a wave buoy has been confirmed in past studies, as already introduced. However, in the Bayesian method, the motion transfer functions are needed which, in turn, means that the detailed hull geometry as well as knowledge about the exact operational condition (e.g., speed and loading condition) of the ship must be available; otherwise, reliable and accurate motion transfer functions cannot be computed resulting in compromised wave spectrum estimates. Initially, we will therefore introduce two sets of results for underscoring the motivation and relevance of a hybrid framework of the WBA, emphasising that we consider exclusively *un*conditioned wave spectrum estimates of the Bayesian method in this preliminary assessment. Afterwards, the hybrid framework will itself be assessed using in-service data from a container ship.

### Preliminary assessment (unconditioned wave spectrum estimates)

In the preliminary assessment, the first set of results is produced by analysis of dedicated full-scale experimental data obtained during a sea trial campaign using a research vessel of the Norwegian University of Science and Technology (NTNU). The second set of results originates from a container ship which had its wave-induced motions measured during nearly two years of service on a North Atlantic route between Europe and Canada.

#### Sea trial experiments

**Figure 4:**
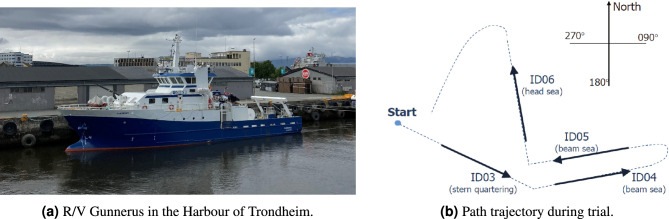
The research vessel Gunnerus is owned and operated by NTNU. Data from a test campaign is studied in the preliminary assessment of the WBA.

Full-scale motion measurements have been collected during sea trials in 2013 with R/V Gunnerus, see Fig. 4. Originally, the sea trials were made to document the effect of a thruster retrofit^38^, and, as part of this, seakeeping runs were made, where one set of run paths, as illustrated in Fig. 4b, is studied herein. The input to the estimation algorithm, i.e. the Bayesian method, is the measured motion components heave, roll, and pitch, as well as the corresponding transfer functions. The transfer functions have been computed with a 2D strip theory code, ShipX^39^, using the exact hull geometry and the precise loading condition and speed of the ship during the trial. On each run path, indicated by IDs 03-06, course and engine power were held constant to ensure stationary conditions, and the durations of the single straight-line runs were 25–30 min, meaning that any involved stochastic process, in these periods, is assumed to have a probability distribution that does not change when shifted in time. During the seakeeping tests, the wave spectrum was measured continuously by a free-floating wave buoy (Datawell BV, Waverider SG) deployed in the test area; but without information about the exact distance between the buoy and the individual run path.

**Figure 5:**
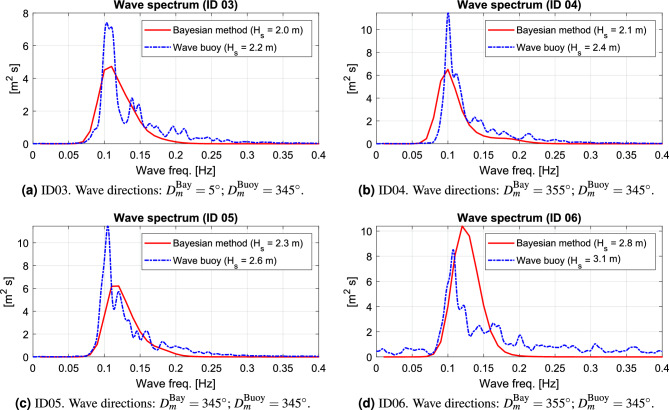
Unconditioned Bayesian wave spectrum estimates obtained by analysis of dedicated sea trial experiments with a research vessel, cf. Fig. 4.

Figure 5 compares wave spectrum estimates by the Bayesian method and corresponding estimates by the wave buoy, noticing that the comparison is made for the point spectra which are integrated versions of the directional wave spectra. The caption of each subplot contains information about the mean wave direction $D_m$, which indicates the compass direction where the waves *come from* following the meteorological convention, refer also to Fig. 4b. Moreover, the significant wave height $H_s$ appears in the legend of each subplot.

Overall, good agreements can be observed from the comparisons in Fig. 5, although Case ID06 stands slightly out compared to the other three cases. In this sense, the main take-away from this initial assessment is that good wave spectrum estimates can be expected, when the WBA is applied under “controlled” conditions. In fact, similar observations have been made in several other studies, where (fully) dedicated experimental datasets, including model tests, are analysed^16,18,24,40–43^. Note that by *controlled conditions* should be understood that the detailed hull geometry is available for computation of the transfer functions, and, furthermore, that the uncertainties in operational parameters (i.e., ship speed and loading condition) are small. This means that the computed transfer functions are reliable and (should) provide a realistic match to the real ship’s motion dynamics in waves, within the implicit assumptions (small-amplitude waves) for using transfer functions computed by linear, potential flow strip theory. Nevertheless, roll is a response dominated by nonlinear effects that cannot be captured using transfer functions, where a linear theory is assumed^44,45^. In this respect, it is noteworthy that the literature^7,8^ includes discussions about replacing roll with sway in the WBA.

As a final comment, it is important to stress that uncertainties are associated not only with the wave spectra of the WBA but also with those of the wave buoy, although the comparison in Fig. 5 indicates a fair agreement. Furthermore, the inherent randomness, in time and space, of ocean waves means that one never truly compares apples with apples, since the ship and buoy are not exactly at the same position. In other words, speaking about a *ground truth* makes little sense in this context.

#### In-service data

**Figure 6:**
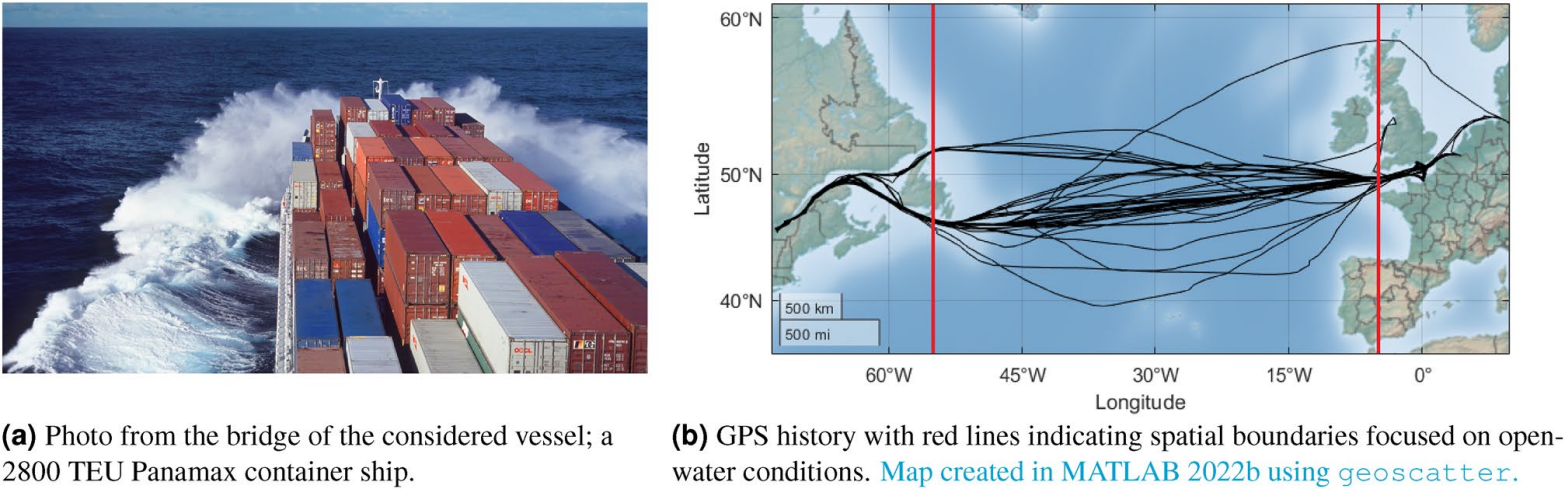
In-service data from a container ship has been analysed. In total, 7644 time series samples of 25 minutes duration have been studied.

**Figure 7:**
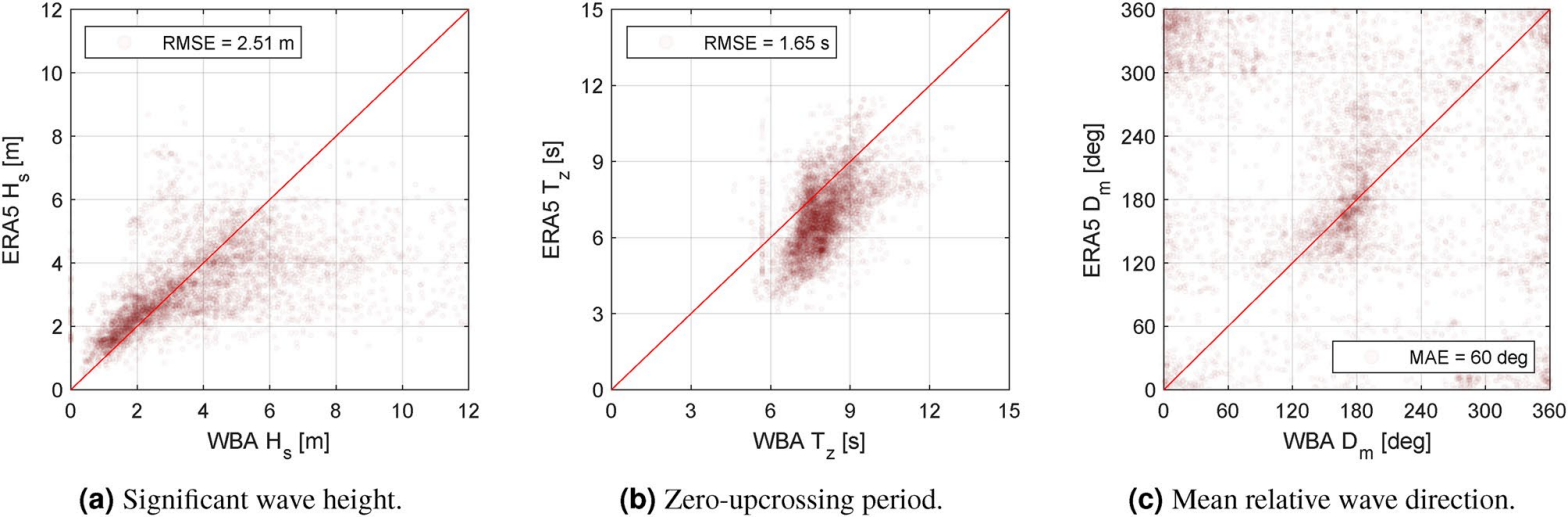
Comparison of integral wave parameters ($H_s$, $T_z$, and $D_m$) from the ERA5 database and corresponding parameters derived from the *un*conditioned wave spectrum estimates by the WBA. The legends inform about the root-mean-squared error (RMSE) for $H_s$ and $T_z$, respectively, and the minimum-absolute error (MAE) for $D_m$.

The second analysis of this preliminary assessment is made with a dataset from the container ship shown in Fig. 6a, noticing that the analysis considers nearly two years of operational data from service on the North Atlantic, see Fig. 6b. The ship and the complete dataset are thoroughly described in the literature^46^. For the present study, it suffices to note that the wave-induced accelerations of one angular motion (pitch) and two translational motions (sway, heave), respectively, are simultaneously used as input to the Bayesian estimation method. The particular dataset does not contain information about the ship’s loading condition (i.e., draught and trim) on the voyages, and, due to the nature of the measurements (in-service data), neither has it been attempted to ensure that the vessel’s speed and course are constant during the data recordings used for wave estimation. Yet, transfer functions *are* needed for the Bayesian method, and a simple solution to account for the missing information with respect to the exact draught is simply to assume a “typical transit draught”^46^ when computing the transfer functions; even though the ship’s real draught and trim may have been (very) different on many of the studied voyages. Thus, it is appreciated that the transfer functions have been computed from the detailed hull geometry, made available to the authors for the particular purpose, and using a commercial software^47^. However, the lack of exact knowledge about the operational parameters (draught, trim, speed, course) and their possible fluctuating behavior, at times, clearly introduce large uncertainties in the computed transfer functions.

Based on the vessel’s GPS history, reanalysis data (hindcast) of integral wave parameters has been obtained from the ERA5 database^12,48^. Specifically, ERA5 estimates of significant wave height ($H_s$), zero-upcrossing period ($T_z$), and mean wave direction ($D_m$) are studied and used as a means for comparing estimates of the WBA using the Bayesian method, repeating that, initially, unconditioned results are considered.

Figure 7 presents the comparison of estimates by the Bayesian method (’WBA’) against the corresponding estimates from ERA5; in the scatter plots, any perfect agreement is reflected by points located on the line of identity (full red line). To assess quantitatively the deviation between corresponding pairs of estimates, root-mean-squared-errors (RMSE), in dimensional form, are also computed and included for $\{H_s, T_z\}$, while the minimum-absolute-error (MAE) is included for $D_m$, accounting for the directional ambiguity. In short, it is evident from the comparison that significant scatter appears, although the markers for each of the three parameters {$H_s$, $T_z$, $D_m$} to some degree distribute around the line of identity.

### Wave spectrum estimates from a hybrid framework

In the hybrid approach, outcomes of a convolutional neural network (CNN) act as constraints on the directional wave spectrum produced by the Bayesian method. The assessment of the hybrid approach is made with the same dataset as presented in the previous subsection focused on in-service data from a container ship. The training of the CNN is based on 80% of the total (filtered) dataset and comprises $K_{t}=3823$ samples^32^, while the validation set makes up the remaining 20% of the data, which means that $K_{v}=956$ samples are left as unseen data. The reader is reminded that a *sample* in this context consists of a 25-minute set of MRU (motion response unit) time series and the corresponding ERA5 wave parameters in terms of {$H_s$, $T_z$, $D_m$}.

**Figure 8:**
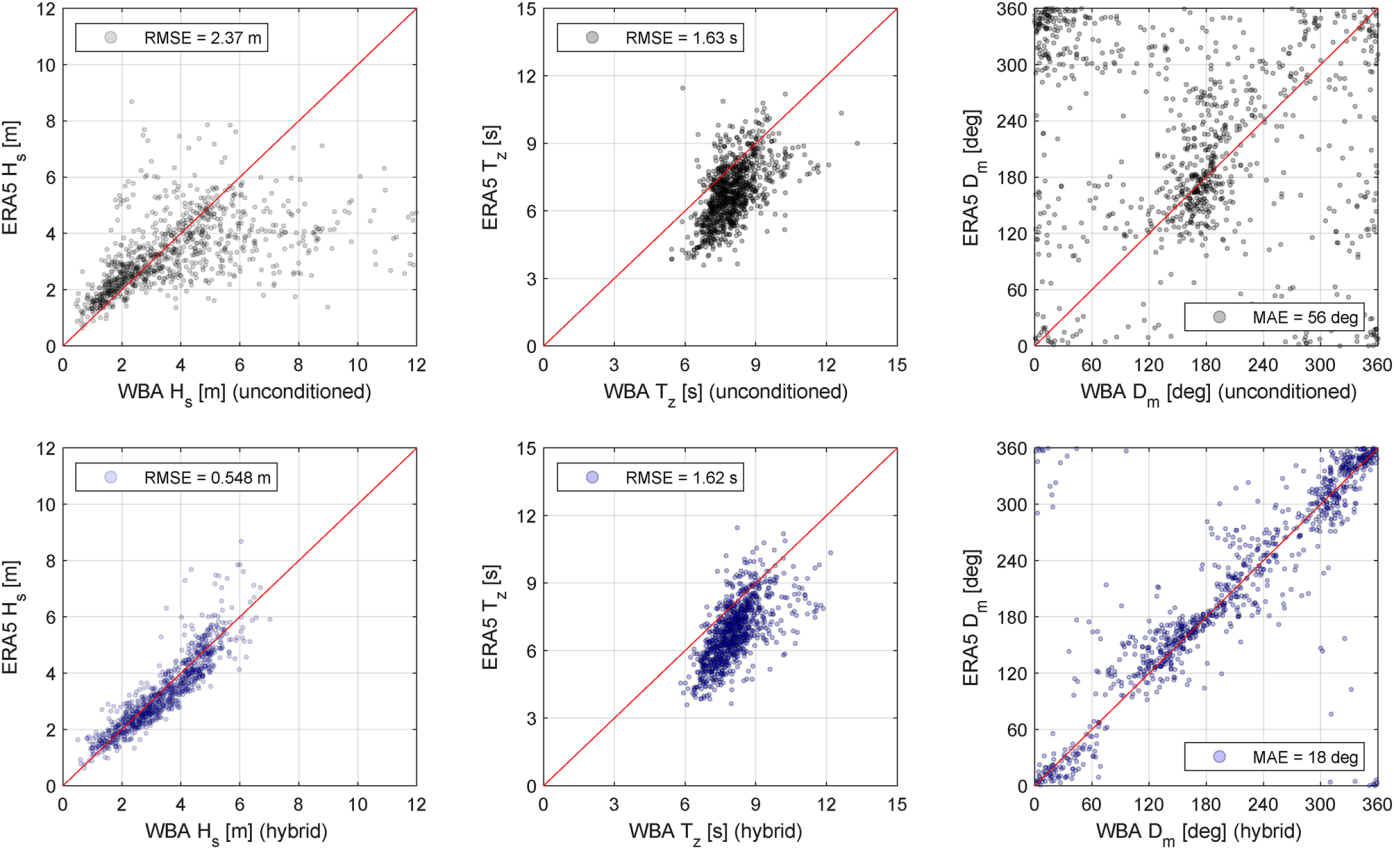
Integral wave parameters from the ERA5 database against corresponding parameters derived from, respectively, the unconditioned wave spectrum estimates by the WBA (top row) and the conditioned estimates using the hybrid framework of the WBA (bottom row). Left-side plots: $H_s$, middle plots: $T_z$, right-side plots: $D_m$.

Figure 8 shows the improvement in accuracy by applying the hybrid framework. In the figure, the three top-plots (unconditioned WBA) should be compared against the three bottom-plots (hybrid WBA); noticing that each sub-plot presents the comparison between estimates of ERA5 and the WBA, similar to Fig. 7, in all cases considering samples of the validation data exclusively. The improvements in estimates of significant wave height ($H_s$) and wave direction ($D_m$) are evident. On the other hand, no notable effect appears on the zero-upcrossing period ($T_z$). The explanation for this observation is that the conditioning of the WBA wave spectrum estimates, using the hybrid framework, is introduced solely through constraints on $H_s$ and $D_m$, as a deliberate choice for the reason discussed further below. It is important to emphasise that the conditioning of the wave spectrum estimates is made using outcomes of a machine learning method (i.e. the CNN) and *not* directly the ERA5 parameters themselves. The reason is that for the hybrid framework to be applicable in real-time, the “constraining parameters” must be immediately available, for instance through a pre-trained machine learning method which itself can be formed by earlier offline training with a proxy for the ground truth, like the ERA5 database.

**Figure 9:**
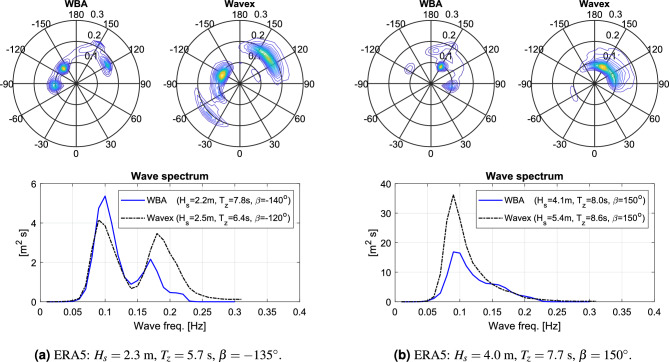
Directional wave spectra by the WBA and a wave radar system (Wavex), and with the corresponding point spectra below. In subplots (**a**) and (**b**), different time stamps (UTC) are considered (**a**: 24-09-2007 00:00; **b**: 06-03-2008 19:00), and the respective sub-captions inform about the corresponding ERA5 wave parameters for the given cases, including the wave encounter angle $\beta$.

While the assessment of the hybrid framework is made entirely through wave *parameters*, it is important to stress that many problems of wave-structure interaction require the directional wave spectrum as the fundamental input, not just the wave parameters. Indeed, the proposed hybrid (“machine learning-informed physics-based”) method provides this, as can be seen from two arbitrarily selected samples in Fig. 9. In fact, in the given cases, the WBA spectra are compared with corresponding directional spectra estimated by a wave radar system; noticing that the ship, in the past, was used as a test platform in a research project about hull structure integrity and, for this reason, the ship was also equipped with a wave radar (Wavex$^{\circledR }$ by Miros), capable of providing estimates of the onsite directional wave spectrum. In this context, to repeat the general message, it is underlined that wave radars come with uncertainties in their estimates as well. Detailed discussions are beyond the present scope, but particular concerns of wave radars relate to, e.g., the accurate estimation of the significant wave height^49^ and the effect of rain clutter^50^.

## Discussion

Inherently, the use of transfer functions, as in the physics-based variants of the WBA, is associated with several uncertainties. It can be difficult to assign single and general causes for the uncertainties, however, the lack of detailed and exact information with regard to operational conditions (draught, trim, speed, etc.) is a primary source when in-service data is studied. In addition, the complex geometry of a ship hull can lead to difficulties in correctly modelling the physics of the motion dynamics of a ship in waves; especially considering higher and steeper waves. What is more, sometimes the hull geometry is not available to the ship operator, in which case so-called closed-form expressions^51,52^ can be used as an approximate alternative for the transfer functions. Nevertheless, transfer functions presumably yield a qualitatively correct picture of a ship’s response in waves. A machine learning model, on the other hand, can infer nonlinearities and other unseen tendencies in the data not captured by transfer functions. By forming a hybrid framework, we have therefore tried to include the best from two fundamentally different methods to reach an approach with the potential to emphasise the pros and minimise the cons of the two respective frameworks being merged.

In the assessment using in-service data, wave parameter estimates from the ERA5 database were considered; both for the direct comparison with output of the WBA and for the training of a convolutional neural network, where the ERA5 parameters acted as a *proxy* for the ground truth. In this sense, the particular database, or a similar database produced by third-generation spectral wave models, can itself be used for making assessments of ship operations, considering aspects of safety as well as performance (fuel consumption analysis, etc.). One disadvantage, however, is that such databases cannot be used for on-board, real-time assessments in the guidance of the shipmaster, since the database is the result of reanalyses made by assimilating the spectral wave model with measured data in offline *post*-processing. Moreover, reanalysis data will practically never be truly applicable to the exact spatio-temporal position of the ship, due to the fixed resolution of the grid in the reanalysis data. On the other hand, the advantage in training the machine learning model with ERA5 is that this approach does not require additional/extra hardware, noticing that a wave radar system needs a dedicated X-band radar for its functional mode of operation.

In connection with the preceding and with a remark about the practical application, it is imagined that the hybrid method could run by introducing an initial ’training phase’. As an example: At day 0 until, say, day 100, wave spectrum estimates are produced by only the Bayesian method (being solely physics-based). From day 101, enough training data (i.e., recorded motions plus ERA5) has presumably been collected for the machine learning model to produce reliable output, and the hybrid method could start producing results, though with continued training of the machine learning model. On the same note, it is important to mention that a new training phase is needed when/if a new ship is considered. That is, results from one ship cannot be transferred directly to another ship; unless the two ships are identical in their design and with similar operational profiles.

In the hybrid approach, the conditioning of the wave spectrum estimates was made by imposing constraints on $H_s$ and $D_m$, but not $T_z$. This is a choice made since the (low-pass) filtering effect of a ship generally implies that high-frequency wave components are not “sensed”, resulting in lost information about the tail of the wave spectrum; with a deteriorating tendency the larger the ship relative to wave length. Consequently, it is thought that it potentially will harm more than it will do good to impose (also) a constraint on $T_z$, since the estimated *conditioned* wave spectrum may end up being distorted, or in other ways nonphysical. In fact, the hypothesis was tested and confirmed on a few samples, although not shown herein, but it will take more thorough investigations to make definitive conclusions.

Somewhat related to the previous point, it should be noted that different types of responses exhibit different (low-pass) frequency characteristics. For instance, in relative terms, motion components like heave, roll, and pitch are typically less excited compared to structural hull girder responses such as bending and twisting, when the ship operates in short waves. In other words, the negative effects of a ship’s low-pass filtering characteristics can be diminished by introducing responses not affected by the low-pass behaviour to the same degree as motion components^10,19,25^. Consequently, designing or selecting the right measurements for improved estimation capabilities would be nice to have as a standard approach, thinking in the direction of a dynamical and automatic selection^53,54^.

As pointed out earlier, it has little meaning to speak about a *ground truth* when measuring ocean waves. Nevertheless, in machine learning, a proxy thereof is needed. In this paper, the ERA5 database has been used, without touching the fact that the database represents *estimates* and is associated with uncertainties itself. In fact, it is possible to include uncertainties in the database from observations, sea surface temperature, and from model-physical parameterisations^55^. Clearly, it could be interesting to consider this kind of uncertainty in the hybrid method, in an extended and future work, as such an extension could make the WBA even more appealing from a practical-application point of view. In this context, it should be noted that how accurate the wave height, for instance, needs to be, depends on the use-case; in some cases a low accuracy is fine, while in other cases we would like high accuracy. On the same note, it is also interesting to mention that a physics-based version of the WBA, making it possible to include the uncertainty in the transfer functions of the ship, has been developed^26^. In the future, it appears interesting to investigate possible combinations of that version with the hybrid framework discussed in the present paper.

It is possible to obtain the detailed directional wave spectrum from the ERA5 database. As already noted, research^30,33,34^ has been initiated for the development of machine learning models capable of reconstructing actual (directional) wave spectra from measured ship responses. The challenge, however, is that wave spectra can be considered as sparse matrices and their prediction is difficult and inefficient. Additionally, larger input/output dimensions lead to a high number of trainable parameters, which makes the models fragile and susceptible to overfitting. Hence, a dimensionality reduction procedure^56,57^ as pre-processing could be a work-around for increasing model efficiency as well as accuracy, like discussed by one study^58^. Although the existing studies aimed at predicting the full wave spectrum appear yet as immature, since they consider simulated data only and/or do not assess the model performance on out-of-sample data and thus reflect reduced credibility, it is believed that successful developments will be presented, sooner or later.

**Figure 10:**
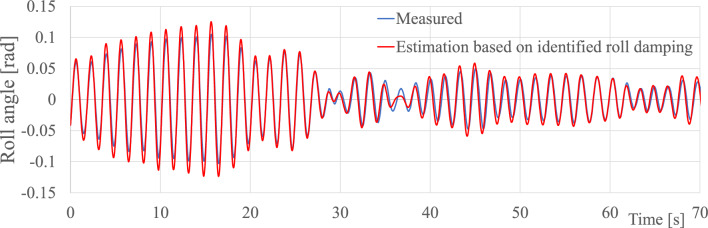
Measured and estimated roll motions in long-crested irregular waves based on model-tests carried out by National Maritime Research Institute (Japan)^59^. The time axis is in the scale of the model (1:72).

For about two decades, we have investigated concepts for estimating waves via measured ship responses, and the technology provides good estimates of the directional wave spectrum compared to other means. Wave spectra are fundamental inputs to the assessment of many wave-structure interaction problems. However, certain types of problems are dominated by nonlinearities, like the prediction of ship rolling and whipping-induced hull girder vibrations, and, in such cases, actual time series sequences of the surface (wave) elevation are needed. Recently, a method for reconstructing the incident wave profile has been proposed^25,43^, and, using this method, a framework for nonlinear roll damping identification has been developed^59^. So far, the framework has been assessed with simulated and model-test data only, see Fig. 10, and further validations with in-service data are thus needed. Another important work for the future will be to investigate the simultaneous use of multiple ships, in a network-based approach, for improved wave estimation *and* forecasting capabilities over larger geographic regions, with repeated reference to Fig. 2 highlighting the potential in using ships as sailing wave buoys. Initial studies in this direction have commenced^60^, including related work focused on deriving spatial wave data from a network of buoys and ships^61^.

## Theory and methods

### Wave parameters and statistical metrics

Wave parameters characterising a directional wave spectrum can be derived by integration^62^. Thus, significant wave height $H_s$, zero-upcrossing period $T_z$, and mean wave direction $D_m$ are all derived from the directional wave spectrum $E(\omega ,\mu )$,

1 $$\begin{aligned} H_s= & {} 4\sqrt{m_0} \end{aligned}$$

2 $$\begin{aligned} T_z= & {} 2\pi \sqrt{\frac{m_{0}}{m_2}} \end{aligned}$$

3 $$\begin{aligned} {D_m}= & {} \text {atan}(d/c) \end{aligned}$$

where

4 $$\begin{aligned} m_n= & {} \int _{0}^{\infty } \omega ^n F\left( \omega \right) d\omega \quad n=\{0,2\} \end{aligned}$$

5 $$\begin{aligned} F\left( \omega \right)= & {} \int _{-\pi }^{\pi } E\left( \omega ,\mu \right) d\mu \end{aligned}$$

6 $$\begin{aligned} d= & {} \displaystyle \int _{-\pi }^{\pi }\int _{0}^{\infty } E\left( \omega ,\mu \right) \sin (\mu ) d\omega d\mu \end{aligned}$$

7 $$\begin{aligned} c= & {} \displaystyle \int _{-\pi }^{\pi }\int _{0}^{\infty } E\left( \omega ,\mu \right) \cos (\mu ) d\omega d\mu \end{aligned}$$

with $\omega$ as the circular frequency in [rad/s] along the wave direction $\mu$. Note that the *wave encounter angle* $\beta$, providing the wave direction relative to the ship, is directly derived from the mean wave direction $D_m$ and the course of the ship $\chi$,

8 $$\begin{aligned} \beta = 180^\circ - (D_m-\chi ) , \qquad if(\beta >180, \beta =\beta -360^\circ ) \end{aligned}$$

following the convention that head waves is $\beta =180^\circ$ and waves approaching from starboard side and port side, respectively, are indicated by ’+’ and ’$-$’, while following waves is $\beta =0^\circ$.

To assess quantitatively the deviation between corresponding pairs of wave parameter estimates, root-mean-squared-errors (RMSE), in dimensional form, are computed for $\{H_s, T_z\}$, while the minimum-absolute-error (MAE) is computed for $D_m$, accounting for the directional ambiguity. The expressions follow from:

9 $$\begin{aligned} \displaystyle \text {RMSE} = \sqrt{\frac{1}{K} \sum _{k=1}^K ({y}_{k,WBA}-{y}_{k,ERA5})^2} \end{aligned}$$

10a $$\begin{aligned} \displaystyle \text {MAE}&= \frac{1}{K}\sum _{k=1}^K \min \{\varepsilon _k,(360^\circ -\varepsilon _k)\} \end{aligned}$$

10b $$\begin{aligned} \displaystyle \varepsilon _k&= |{D}_{m,k,WBA}-{D}_{m,k,ERA5}| \end{aligned}$$

where *y* indicates an estimate of the particular parameter in question, i.e. $\{H_s, T_z\}$, for sample *k* considering WBA or ERA5, indicated by indices.

### Wave-to-motion transfer functions

In the most basic sense, *transfer functions* inform how a ship responds to regular (harmonic) waves of unit amplitude. The ship’s linear response in real (irregular) waves can be found by combination (i.e. multiplication) of the transfer functions and the wave spectrum describing the real waves. The interested reader will find several excellent textbooks on the topic^44,45,62–64^.

### Computation of response spectra

To construct the cross spectra, needed as input to the WBA, FFT is performed using the Matlab function cpsd, which is based on Welch’s averaged, modified periodogram method applied on eight sections with 50% overlap, and with each section windowed with a Hamming window^65^.

### The wave buoy analogy (WBA)

#### Bayesian method

A thorough account of the Bayesian method is given in the literature^37,66^, and the particular implementation made in this paper considers ships with forward speed^8,13^. The following contains an introduction to only the most central aspects. From a measured set of responses, corresponding response spectra $S_{i,j}(\omega _{e,l})$, $i,j = \{z,\phi ,\theta \}$, can be computed under the assumption of stationary and ergodic processes. Here, $\omega _{e,l}$ defines the encounter frequency discretised by $l = 1,2,...,L$ components. Formally, the directional wave spectrum $E(\omega , \mu )$ can be determined from the spectral equation,

11 $$\begin{aligned} \displaystyle \min \sum _{i,j} \sum _{l=1}^{L}\left| {S}_{i,j}(\omega _{e,l}) - \int _{0}^{2\pi } \left\langle \Phi _i(\omega ,\beta )\overline{\Phi _j(\omega ,\beta )} E(\omega ,\mu )\frac{d\omega }{d\omega _e} \right\rangle _{\omega _{e,l}} d\mu \right| ^2 \end{aligned}$$

where the transfer function is $\Phi _i(\omega ,\beta )$, and the overline denotes the complex conjugate. The intrinsic frequency is $\omega$ [rad/s], and $\beta = (180 + \chi - \mu$) [deg] is the wave encounter angle with waves propagating *from* the compass direction $\mu$ while the compass heading of the ship is $\chi$. The triangular brackets $\left\langle \cdots \right\rangle _{\omega _{e,l}}$, with $\omega _{e,l}$ as index, are used to emphasise that evaluation happens for a given frequency of encounter. This requires consideration of the Doppler effect^63,67^, relating the intrinsic frequency to the encounter frequency (or vice versa) via the wave encounter angle and ship speed, respectively. The expression in the triangular brackets is thus the (theoretically) computed response spectrum converted to the encounter domain. While the formal notation in Eq. (11) excludes details about the actual implementation, the specific literature^8,13^ includes these, and can be accompanied by a pseudo-algorithm^68^ for ease of practical implementation. It is noteworthy that ship speed, for matters of fuel performance, will normally be held constant in service conditions, excluding all maneuvering operations, e.g., related to departures and arrivals in harbours. Thus, for most cases in service conditions, the variation in ship speed relative to the average value over, say, 25-30 minutes is insignificant. The Bayesian method solves for the unknown wave spectrum $E(\omega , \mu )$ at all discrete pairs of $(\omega ,\mu )$, necessitating regularisation for dealing with the highly under-determined equation system. As such, the method relies on the inclusion of *prior information* to regularise the solution of the governing equation, cf. equation (11). Specifically, constraints are set on the second order derivative, controlling the smoothness of the mathematical surface describing the directional wave spectrum. In summary, it can be said that the Bayesian method establishes a wave spectrum obtained as a compromise between the agreement between the data and the theoretical prediction, formally controlled by Eq. (11), and the smoothness of the wave spectrum. To “guide” the compromise, an objective criterion (referred to as ABIC) is introduced, as carefully explained in the specific literature^8,13^.

#### Convolutional neural network

Neural networks can produce a satisfactory mapping from measured vessel responses to wave parameters. In this paper, the *sea state identification*, to differentiate from wave spectrum estimation where the entire directional wave spectrum is obtained, builds on an implementation of an Inception model^69^. Specifically, the identification algorithm^32^ makes use of response spectra, applied in a multi-task learning (MTL) setting^70^, for identification of significant wave height, peak period, and wave direction. In MTL, each output is considered as a separate task with its own dedicated branch of fully connected hidden layers and corresponding output layers. Such an architecture is selected to favour physical interdependencies between the wave parameters and improve the model’s generalisation capability^71^. In the current work, the ship’s speed and draught were not fed into the model; draught was not measured, thus “unknown”, and the speed was relatively constant, varying in the range of 18-20 knots for the vast majority of the data^32^. In case of more varied operational conditions, however, it will be necessary to refine the neural network, for instance by considering a multi-modal convolutional neural network, which is characterised by several input branches and discussed in greater detail in the literature^32^.

#### Hybrid framework

In the hybrid framework^11^, output ($H_s$ and $D_m$) from the convolutional neural network are considered as physical constraints on the directional wave spectrum to be estimated. This means that additional equations are concatenated to the governing equation system represented by Eq. (11). Specifically, the following equations are considered:

12a $$\begin{aligned} \displaystyle \int \int \widetilde{E}\left( \omega ,\mu \right) d\omega d\mu = \frac{1}{16}\widehat{H}_s^2 \end{aligned}$$

12b $$\begin{aligned} \displaystyle \int \int \widetilde{E}\left( \omega ,\mu \right) \sin \mu \ d\omega d\mu = \widehat{d} \end{aligned}$$

12c $$\begin{aligned} \displaystyle \int \int \widetilde{E}\left( \omega ,\mu \right) \cos \mu \ d\omega d\mu = \widehat{c} \end{aligned}$$

where Eqs. (12b) and (12c) in combination relate to the mean relative wave direction, cf. Eq. (3). It is understood that the wide-tilde, in $\widetilde{E}(\omega ,\mu )$, is used to indicate the directional wave spectrum to be estimated, whereas the hat-notation on the right-hand side of the equations is used to indicate that the parameters are known from another source; in this case, they are the output from the machine learning model, i.e. the convolutional neural network. Details of the hybrid framework are presented in^11^.

### ERA5 database

Atmospheric reanalyses provide estimations of coherent records of global air-sea-land circulation^72–74^. Focusing on sea surface conditions, the spatio-temporal development of ocean wave systems can be described by the energy balance equation^75–78^. Specifically, using third-generation spectral wave models, the time- and position-dependent wave spectrum is computed by integration of the energy balance equation. In addition, observations of wind and waves are assimilated. The ERA5 database^12,48^ is the result of this type of modelling, and ECMWF (European Centre of Medium-Range Weather Forecasts) has made the reanalysis freely available through EU funding^79^. In the database, hourly ocean wave data on single levels is available on a regular latitude-longitude grid at $0.5^{\circ } \times 0.5^{\circ }$ resolution, and interpolation to the exact ship position is made accordingly^80^.

## Data Availability

The dataset from R/V Gunnerus is not publicly available due to an agreement between NTNU and Rolls-Royce Marine but is available from the authors on reasonable request. The in-service data that supports the findings of the hybrid method is available from DNV but restrictions apply to the availability of this data, which was used under license for the current study, and so is not publicly available. Data is however available from the authors upon reasonable request *and* with permission of DNV.
